# Electroacupuncture Increases the Hippocampal Synaptic Transmission Efficiency and Long-Term Plasticity to Improve Vascular Cognitive Impairment

**DOI:** 10.1155/2022/5985143

**Published:** 2022-06-23

**Authors:** Yaling Dai, Yuhao Zhang, Minguang Yang, Huawei Lin, Yulu Liu, Wenshan Xu, Yanyi Ding, Jing Tao, Weilin Liu

**Affiliations:** ^1^College of Rehabilitation Medicine, Fujian University of Traditional Chinese Medicine, Fuzhou, Fujian 350122, China; ^2^Rehabilitation Industry Institute, Fujian University of Traditional Chinese Medicine, Fuzhou, Fujian 350122, China; ^3^Fujian University of Traditional Chinese Medicine, Fuzhou, Fujian 350122, China

## Abstract

Studies have shown that electroacupuncture (EA) can effectively improve vascular cognitive impairment (VCI), but its mechanisms have not been clearly elucidated. This study is aimed at investigating the mechanisms underlying the effects of EA treatment on hippocampal synaptic transmission efficiency and plasticity in rats with VCI. *Methods*. Sprague–Dawley rats were subjected to VCI with bilateral common carotid occlusion (2VO). EA stimulation was applied to Baihui (GV20) and Shenting (GV24) acupoints for 30 min once a day, five times a week, for four weeks. Our study also included nonacupoint groups to confirm the specificity of EA therapy. The Morris water maze (MWM) was used to assess cognitive function. Electrophysiological techniques were used to detect the field characteristics of the hippocampal CA3–CA1 circuit in each group of rats, including input-output (I/O), paired-pulse facilitation ratios (PPR), field excitatory postsynaptic potential (fEPSP), and excitatory postsynaptic current (EPSC). The expression of synapse- and calcium-mediated signal transduction associated proteins was detected through western blotting. *Results*. The MWM behavioural results showed that EA significantly improved cognitive function in VCI model rats. EA increased the I/O curve of VCI model rats from 20 to 90 *μ*A. No significant differences were observed in hippocampal PPR. The fEPSP of the hippocampal CA3–CA1 circuit was significantly increased after EA treatment compared with that after nonacupuncture treatment. We found that EA led to an increase in the EPSC amplitude and frequency, especially in the decay and rise times. In addition, the protein expression and phosphorylation levels of N-methyl-D-aspartate receptor 2B, *α*-amino-3-hydroxy-5-methyl-4-isoxazole propionate receptor 1, and Ca^2+^-calmodulin-dependent protein kinase II increased to varying degrees in the hippocampus of VCI model rats. *Conclusion*. EA at GV20 and GV24 acupoints increased the basic synaptic transmission efficiency and synaptic plasticity of the hippocampal CA3–CA1 circuit, thereby improving learning and memory ability in rats with VCI.

## 1. Background

Vascular cognitive impairment (VCI) is a cognitive impairment syndrome caused by chronic cerebral tissue ischemia and hypoxia. Clinically, it can develop from mild cognitive impairment to vascular dementia, which is the second most common dementia after Alzheimer's disease and brings a great burden to the social medical system [1]. VCI is mainly manifested as a decline in learning and memory ability [2]. Its pathological mechanisms include the reduction of synaptic proteins, damage to synaptic plasticity [3], impaired cholinergic system [4], excitotoxicity [5], oxidative stress, inflammation [6], and genetic factors [7]. However, no effective treatment is currently available. Recently, studies have shown that electroacupuncture (EA) can effectively improve VCI. The hippocampus is a key structure of the brain involved in learning and memory, which mediates their storage by changing the synaptic interaction [8]. In VCI model animals, the hippocampus is vulnerable to damage induced by hypoxia and ischemia, involving significant hippocampal atrophy [9], neuron ultrastructure damage in the hippocampus [10], oxidative stress [11], and impaired expression of synapse-related proteins and synaptic plasticity [12].

Synaptic morphology and structural plasticity are the material bases for functional synaptic plasticity. Long-term potentiation (LTP) is generally considered a typical cellular electrophysiological model reflecting synaptic plasticity and is widely used to evaluate cognitive functions. Elucidating the mechanisms of maintenance may provide insights into the molecular processes involved in the stability of stored memory. The Ca^2+^-calmodulin-dependent protein kinase II (CaMKII), a highly abundant brain protein concentrated in the postsynaptic density, is strongly implicated in LTP. During LTP induction, Ca^2+^ enters the N-methyl-D-aspartate receptor (NMDAR) and binds to calmodulin. Calmodulin then activates CaMKII, which phosphorylates the GluA1 subunits of the*α*-amino-3-hydroxy-5-methyl-4-isoxazolepropionic acid receptors (AMPARs) and the auxiliary subunit of AMPARs. The first reaction increases the conductance of AMPARs, and the second allows more AMPARs to bind to the synapse through the postsynaptic density protein 95. Together, these processes provide a mechanistic explanation for the early LTP phase, within approximately the first 30–60 min [13].

The use of acupuncture, an ancient globally renowned alternative and complementary branch of medicine, is based on a large body of preclinical and clinical research. Studies are slowly uncovering the mechanisms underlying the protective effects of acupuncture. Many studies have shown that EA can improve learning and memory. Our previous research confirmed that EA at the Baihui (GV20) and Shenting (GV24) acupoints can effectively improve the minimental state examination and Montreal cognitive assessment scores of patients with cerebral ischemia and improve their overall cognitive dysfunction [14]. In animal experiments, EA at the GV20 and GV24 acupoints effectively alleviates learning and memory dysfunction in VCI model rats [15]. Regarding the potential mechanism of EA improving cognitive function of VCI model rats, studies have shown that EA can reduce oxidative stress in the brain [16], relieve neuronal apoptosis [17], slow down neuroinflammation [18], regulate brain glucose metabolism [19], regulate neurotransmitter release [20, 21], and improve cerebrovascular function [22]. Moreover, studies have pointed out that the improvement of most pathological changes is beneficial to the release of neurotransmitters, transmission of nerve signals, and enhancement of synaptic plasticity [23–25]. Therefore, this study is aimed at investigating the effect and underlying mechanisms of the EA treatment on hippocampal synaptic transmission efficiency and plasticity in VCI model rats. Synaptic transmission efficiency and plasticity play key roles in regulating learning and memory and are currently considered as the cellular physiological basis of the two phenomena [26]. The improvement of transmission efficiency and plasticity of synapses may be a direct or indirect impact of acupuncture reversing VCI pathology to improve learning and memory, despite the lack of direct experimental evidence [27]. Therefore, we treated VCI rats with EA for 4 weeks and evaluated them using new object recognition test and Morris water maze, and results showed improved learning and memory functioning. The expression levels of PPR, LTP, I/O curve, sEPSC, and synaptic plasticity-related proteins GluR1, NMDAR2B, CaMKII, and P-CaMKII in the hippocampus increased, indicating an enhancement of the synaptic transmission efficiency and plasticity. Our results suggest that EA can improve learning and memory functioning in VCI rats, possibly by enhancing synaptic transmission efficiency and plasticity in the hippocampus.

## 2. Methods

### 2.1. Experimental Animal

All experimental procedures were strictly in accordance with the International Ethical Guidelines and the National Institutes of Health Guide for the Care and Use of Laboratory Animals and were approved by the Ethics Committee of Fujian University of Traditional Chinese Medicine [SYXK (Min) 2020-0007]. Sixty male Sprague–Dawley (SD) rats (8-week-old; weight, 250–300 g) were purchased from the Shanghai SLAC Laboratory Animal Co., Ltd. (Shanghai, China) [the batch number SCXK(Hu)2012-0002], weighed, and numbered after one week of adaptive feeding at the Experimental Animal Centre of Fujian University of Traditional Chinese Medicine.

### 2.2. VCI Model Establishment

Out of the 60 male SD rats, 12 were randomly assigned to the sham operation group, and the other 48 were selected as models. The bilateral common carotid occlusion (2VO) operation was used to prepare the VCI model, and in the sham operation group, the bilateral common carotid arteries were only separated without ligation [28]. Three-dimensional time-of-flight magnetic resonance imaging (3D-TOF MRI) and novel object recognition tests were used to identify the model.

Thirty-six rats that were successfully modelled and qualified in the operation group were randomly divided into 2-VO, EA, and nonacupoint (Non-acu) groups. The notion of a nonacupoint is obscure, and there may be a role for specific factors of nonacupoints lateral to acupoints. In this study, nonacupoints under the flanks were selected as the nonacupoints for the operation. The acupuncture points are located under the two flanks, which are not on the upper meridians, and are convenient for positioning and needle insertion.

After the rats in each group were anaesthetised, the neck skin was cut longitudinally using surgical scissors, one side of the common carotid artery was separated, and the vagus nerve accompanying the common carotid artery was separated to avoid damage. The common carotid artery was then ligated using a nylon cord. After 5 min, the other common carotid artery was separated and ligated in the same manner. Lastly, the wound was cleaned, skin was sutured, and penicillin was injected. Rats were injected with the same amount of penicillin after suturing and returned to the cage together with the model.

### 2.3. EA Treatment

EA stimulation was applied at the Baihui acupoint (on the middle of the parietal bone, 2 mm obliquely backward) and the Shenting acupoint (on the anterior midline, in front of the frontal-parietal suture junction, 2 mm obliquely backward) with a 0.5 mm needle (No. 30 Hua Tuo Brand), 30 min/time, once a day, five days/week, for four weeks.

The stimulation parameters were set to 6 V, 1–3 mA, sparse-dense waves, and 2/20 Hz, and an electroacupuncture therapeutic apparatus (G6805; Hua Tuo Brand) was used.

The nonacupoint (Non-acu) group used for the nonacupoints (on the lower part of the bilateral flank, 3 mm obliquely downward) was subjected to stimulation with the same parameters. The sham operation and 2VO groups were grasped under the same conditions and then returned to their cages for rearing [29].

### 2.4. 3D-TOF MRI

A 3D-TOF MRI, also known as bright-blood technology, showed cerebral artery stenosis, local vertebrobasilar stenosis, or signal interruptions. A 7.0 T small animal MRI scanner (Germany Bruker Biospec 70/20 USR) was used for the 3D TOF magnetic resonance angiography. All rats were scanned using the same parameters. Each rat was anaesthetised in the supine position with mixed gas containing 3% isoflurane for 5 min and then scanned. In this study, the imaging parameters of the 3D ASL series were the same as those reported in previous studies. Isoflurane (0.2%) was administered through a nasal cannula to maintain anaesthesia. During the scan, an animal physiological detector was used to detect the body temperature and heart rate of the rats, and a temperature control system and ventilation system were used to maintain the stability of the physiological state during the entire experiment.

The 3D-TOF scan parameters were as follows: TR, 15 ms; TE, 2.7 ms; FoV, 30 × 30 × 24 mm; average, 1; and slice thickness, 24 mm [30].

### 2.5. Learning and Memory Behavioural Test

#### 2.5.1. Novel Object Recognition Test

A novel object recognition test was conducted to detect each group of learning and memory function. This system included a 60 cm × 40 cm × 80 cm black plastic open box, two rows of LED light bars on the left and right sides of the box, and 3 solid objects. At the top, a camera was used to observe the activities and exploration process of the animals. The experiment process was divided into 3 stages, the adaptation period, the familiarization period, and the testing period. The first day was adaptation period; the rats were allowed to be familiar with the experiment box for 10 minutes. During the familiarization period, two identical objects were put into the two opposite corners of the experiment box. The total exploration time of the two objects within 5 minutes of the rat was recorded. After an interval of 60 minutes, the rat was allowed to enter the first test period. During the test period, one of the familiar objects was replaced with another novel object of similar size but different shapes and colors, and the time in the exploration of novel and familiar objects was recorded. 24 h later; rats were also put into the box as the same way and allowed to explore freely for 5 min. Discrimination index (DI) was adopted to evaluate the learning and memory function. The discrimination index calculation formula is DI = (*N* − *F*)/(*N* + *F*) *x* 100%, where *N* (novel) is the exploration time of novel objects and *F* (familiar) is the exploration time of familiar objects [31].

#### 2.5.2. Morris Water Maze Test

The Morris water maze test was used to assess cognitive ability. The maze consisted of a 100 cm diameter circular pool filled with water at 25°C and a 7 cm diameter escape platform in the centre of the designated target quadrant, approximately 5 mm below the water level. The rats were then trained and given 90 s to find the platform four times a day for five days. The distance swam, number of crossings, position of the target platform and the other three platforms, and time spent in the quadrants of the four platforms were measured [32].

### 2.6. Electrophysiology

Candidate MSNs in the dorsolateral striatum were identified using infrared differential interference contrast video microscopy (BX50WI; Olympus, Japan). The patch pipettes (3–5 M*Ω*) were made from borosilicate glass capillaries pulled on a P-1000 micropipette puller (Sutter Instruments, Novato, CA, USA). Whole-cell patch clamp recordings were performed in gap-free acquisition mode with a sampling rate of 10 kHz and low-pass filtered at 3 kHz, using a MultiClamp 700B amplifier, Digidata 1550 digitizer, and pClamp 10.6 software (Molecular Devices, Sunnyvale, CA, USA). The access resistance was continuously monitored during the experiments. Cells were excluded if their access resistance was >25 M*Ω*.

Artificial cerebrospinal fluid (ACSF, 1000 mL) containing NaCl, 126; KCl, 2.5; NaH_2_PO_4_, 125; NaHCO_3_, 26; D-glucose, 10; CaCl_2_, 0.5; and MgSO_4_, 10 (in mM) was prepared before the experiment, and 300 mL was frozen for 60 min. The experimental animals were anaesthetised with 5% sodium pentobarbital and immediately decapitated. The rat brain tissue was placed on the plane of the slice slot holder with glue and placed in a tank filled with an ACSF ice-water mixture and oxygen (95% O_2_+5% CO_2_ mixture). Brain tissue was cut into 300–400 *μ*m slices using a Leica vibrating microtome and immediately transferred to an isolated brain slice incubator. After incubating at a constant temperature water bath at 31°C for 30 min, the patch clamp field potential was recorded. When the recording electrode was moved to contact the ACSF, the system was adjusted to the current clamp mode (*I* = 0), the liquid junction potential was adjusted to near 0, and the resistance of the recording electrode was checked. The resistance of the recording electrode was required to be 3–6 *Ω*. The stimulation electrode was stimulated for 1–7 *μ*A, with a 20 s interval stimulation square wave, and the field excitatory postsynaptic potential (fEPSP) was recorded. Once the waveform was stable, the stimulation intensity was changed to record the I/O curve according to the change in stimulation intensity (a total of nine stimulation currents of 10–90 *μ*A were administered three times with a 20 s interval for each stimulation). The 30% stimulus intensity of the recorded maximum peak value of fEPSP was used as the PPR basic field potential stimulus intensity (two stimulus currents were applied at intervals of 10, 20, 50, 100, 200, and 500 ms). In the LTP test, 30% electrical stimulation of the maximum peak value was used as the baseline basic field potential stimulation intensity (stimulation was given once in 20 s, and the baseline was recorded for 20 min. After baseline recording, 100 Hz high-frequency stimulation was given through the stimulation electrode (twice with an interval of 30 s) to induce LTP, basic field potential stimulation was continued, and fEPSP was recorded for 60 min. For spontaneous excitatory postsynaptic current (sEPSC) recording, MSNs were voltage-clamped at −70 mV in the presence of picrotoxin (50 *μ*M). The internal electrode fluid for resting membrane potential and sEPSC recording contained (in mM) K-gluconate, 140; KCl, 3; MgCl_2_, 2; EDTA, 0.2; HEPES, 10; and ATP (Na ^+^ salt), 2 with pH adjusted to 7.2 with KOH and osmotic pressure adjusted to 280–290 mOsmol/L [33].

### 2.7. Western Blotting

Protein concentration was assayed after extraction of bilateral hippocampal tissue protein supernatant with protein lysate. The sample volume for the initial determination of the internal reference was 6 *μ*L per well, the electrophoresis current was kept as constant as possible, and the electrophoresis voltages and time were as follows: 20 V-10 min, 60 V-20 min, and 100 V-60 min, respectively. The polyvinylidene fluoride (PVDF) membrane was removed and placed in triethanolamine-buffered saline (TBS) with Tween 20 (TBS-T) for washing (5 min × 3 times) and was blocked at room temperature (23-26°C) for 1 h. After these steps, the membrane was incubated with primary at 4°C overnight. Then, the membrane was washed and incubated in secondary antibody for 1 hours at room temperature for 1 h. Immunoreactive bands were visualised using the Image Quant LAS 4000 imaging system (Fujifilm USA, Valhalla, NY, USA), and densitometric analyses were performed using Image Gauge analysis software (Fujifilm, USA). The brightness and contrast of the raw blots were equally adjusted across the entire image using Adobe Photoshop CS5 software (Adobe Systems, Ottawa, ON, Canada) to generate representative images [34].

### 2.8. Statistical Analysis

The experimental data were analysed using SPSS 21.0. For normal distribution, results were expressed as the mean ± standard deviation (*X* ± *S*). The experimental results were graphically presented using GraphPad Prism version 6.01. The escape latency of the Morris water maze test, PPR, and I/O curves of electrophysiology were analysed using repeated-measure analysis of variance (ANOVA). Data for novel object recognition, number of crossing platforms, fEPSP slope, and protein levels were analysed using one-way ANOVA. In addition, data were consistent with the homogeneity test, and the least significant difference was used for comparison within groups. Statistical significance was set at *P* < 0.05.

## 3. Results

### 3.1. EA Improved the Spatial Memory Function in VCI Rats

**Figure 1:**
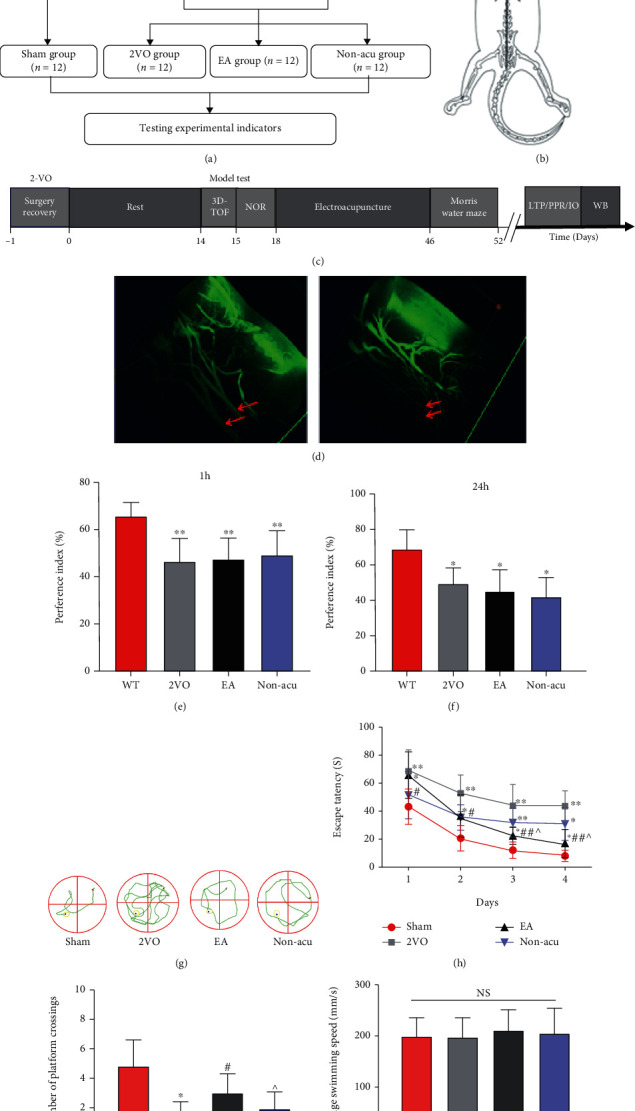
Assessment of MRI and learning and memory behaviours in rats with VCI. (a) Experimental flowchart. (b) Schematic diagram of acupuncture. (c) Experiment timeline. (d) Model group 3D-TOF imaging of bilateral common carotid arteries in the sham operation group indicated via red arrows. (e) New object recognition test (1 h) in each group of rats before the intervention. (f) New object recognition test (24 h) for each group of rats before the intervention. ^∗^*P* < 0.05;  ^∗∗^*P* < 0.01. (g) Typical trajectory diagram for each group in the water maze. (h) Results of water maze escape latency in various groups of rats. (i) Number of times rats crossed the platform in each group. (j) Mean swimming speed for each group of rats. ^∗^*P* < 0.05 and^∗∗^*P* < 0.01, Sham vs. 2VO; ^#^*P* < 0.05, 2VO vs. EA; ^^^*P* < 0.05, EA vs. Non-acu.

The experimental timeline and acupuncture position are shown in Figures 1(a)–1(c). In this experiment, the more commonly used 2VO method was used to prepare the VCI rat model, in which the bilateral common carotid arteries were permanently ligated to cause a chronic state of cerebral hypoperfusion, resulting in ischemic and hypoxic damage to the brain tissue. We detected the presence of bilateral common carotid arteries in the VCI rats and sham operation groups two weeks after surgery using 3D-TOF MRI. MRA further confirmed that the effectively occluded bilateral common carotid arteries presented the absent signal. Moreover, the basilar artery became tortuous after modelling, and rats with normal bilateral common carotid artery after modelling were excluded from the group (*n* = 1) (Figure 1(d)), and the remaining rats were used for behavioural testing of new object recognition. Compared with the sham operation group, in the 2VO, EA, and Non-acu groups, the object preference coefficient decreased significantly at 1 h (shown in Figure 1(e)) and 24 h (shown in Figure 1(f)) (*P* < 0.05), suggesting that VCI rats presented some degree of learning memory dysfunction.

The Morris water maze test showed that in the 4-day learned locomotor navigation experiment in rats (shown in Figure 1(g)), the 2VO group showed a significantly higher latency than that of the sham operation group (*P* < 0.01), suggesting that VCI model rats had impaired spatial learning memory formation functions. The EA and Non-acu groups showed an increase in the duration of the escape latency (*P* < 0.05). The EA group had significantly shorter escape latency than the 2VO and Non-acu groups *P* < 0.01 (shown in Figure 1(h)). The number of platform crossings was significantly reduced in the 2VO, EA, and Non-acu groups (*P* < 0.01). However, the number of rats in the EA group crossing the platform increased (*P* < 0.01; Figure 1(i)), indicating that EA at the GV20 and GV24 acupoints can effectively improve the spatial learning memory function of VCI rats. The mean swimming speed of each group of rats was analysed through one-way ANOVA, and the results showed that the speed of each group was not statistically significant (*P* > 0.05; Figure 1(j)).

### 3.2. EA Rescues Long-Term Potentiation Defects in VCI Rats

**Figure 2:**
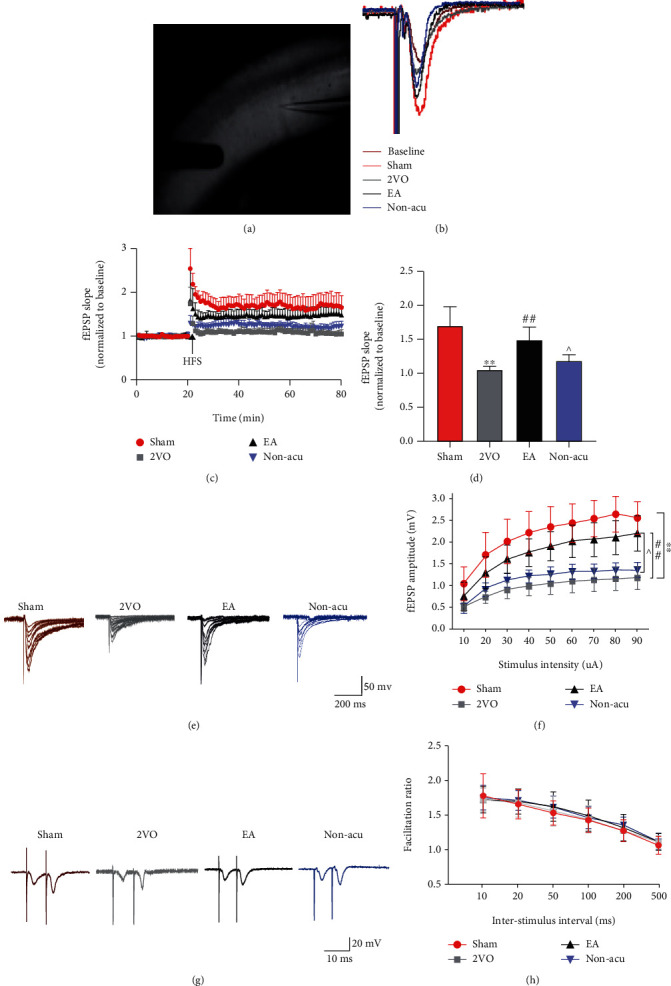
Electroacupuncture improves synaptic plasticity of hippocampal CA3–CA1 neural circuit in VCI rats. (a) Schematic representation of the hippocampal circuit and positioning of the stimulating and recording electrodes for LTP at the Shaffer collateral-CA1 synapses (CA3–CA1 LTP). (b) A schematic diagram showing the generation of LTP. (c) Successful recording of LTP via patch-clamp. (d) Quantification of LTP elicited in the CA3–CA1. (e) The I/O curves for all recorded group are summarized. (f) I/O curve recordings in the CA3–CA1 region of the hippocampus in each group of rats. (g) Representative paired-pulse ratio (PPR) with different interpulse intervals at CA3–CA1. (h) Results of PPR recordings in the hippocampal CA3–CA1 region of rats in each group. ^∗^*P* < 0.05 and^∗∗^*P* < 0.01, Sham vs. 2VO; ^#^*P* < 0.05, 2VO vs. EA; ^^^*P* < 0.05, EA vs. Non-acu.

The analysis of the fEPSP slopes showed a significant difference among the sham operation, 2VO, EA, and Non-acu groups (*n* = 6 per group). High-frequency stimulation (HFS) of the Schaffer collateral inputs to CA3–CA1 pyramidal cells induced stable LTP in the slope of fEPSP in the sham operation group rats (60 min after HFS, 170.61 ± 25.64% of baseline values; Figures 2(a) and 2(b)). In contrast, in the 2VO group, the fEPSP slope was significantly reduced (60 min after HFS, 111.17 ± 16.68% of the baseline values, *P* < 0.01 vs. the sham operation group). However, EA reversed the 2VO-induced LTP impairment (60 min after HFS, 150.00 ± 19.00% of baseline values, *P* < 0.01 vs. the 2VO group). Compared to the EA group, the Non-acu group was reduced (60 min after HFS, 119 ± 0.9% of baseline values, *P* < 0.05, Figures 2(c) and 2(d)).

The input-output curves were constructed by recording the fEPSP amplitude from CA1 following stimulation of Schaffer collaterals with increasing strengths (10–90 *μ*A, *n* = 6 per group). A significant reduction was observed in the I/O curve of the 2VO group compared to that of the sham operation group (*P* < 0.01; Figure 2(e)). Moreover, treatment with EA increased the fEPSP amplitude in the 2VO group compared to that in the EA group (*P* < 0.01). No significant differences were observed between the sham operation and EA groups. Compared with the Non-acu group, the EA group showed an increased fEPSP amplitude (*P* < 0.05; Figure 2(f)).

Short-term plasticity was evaluated using the PPR in interstimulus intervals (10, 20, 50, 100, 200, and 500 ms). We found no statistical difference in PPR 10 ms before HFS delivery in the four groups (Figure 2(g)). This suggests that there was no significant difference in the excitatory presynaptic transmission function of neurons in the hippocampal region of the rats between the groups (Figure 2(h)).

### 3.3. EA Enhanced Excitatory Synaptic Transmission Dynamics in the Hippocampal CA1 Region in VCI Rats

**Figure 3:**
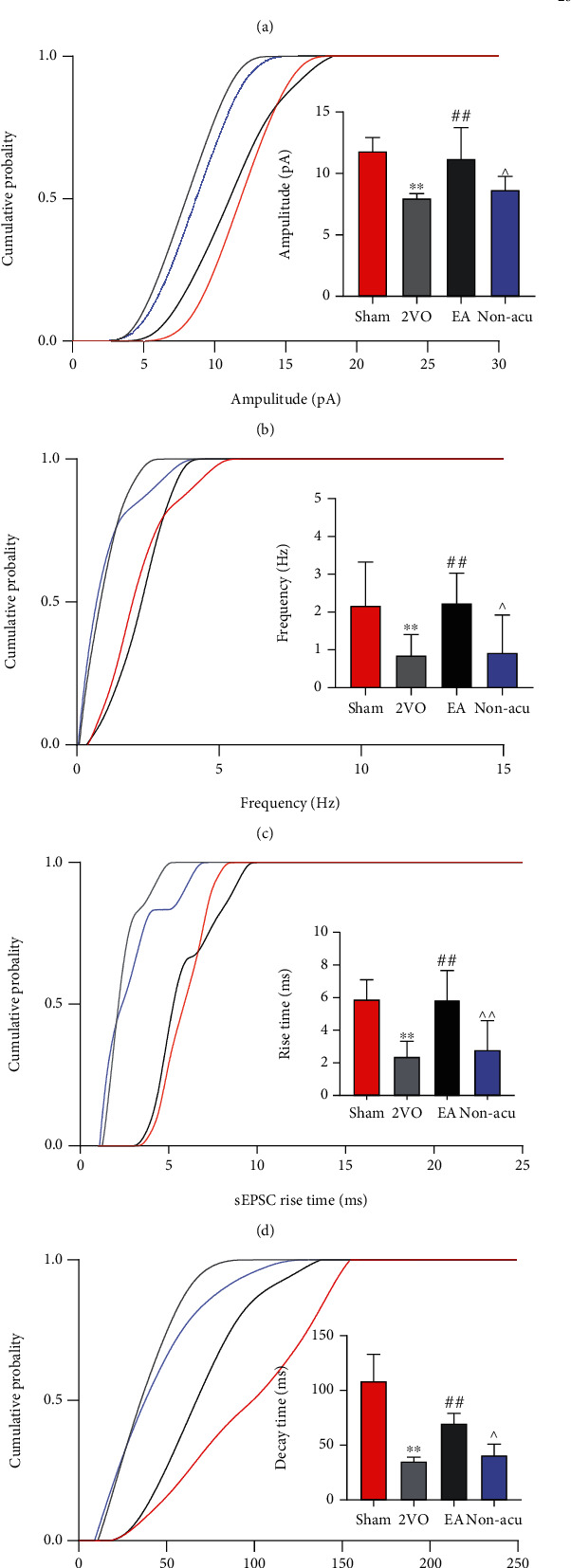
Electroacupuncture improves synaptic transmission rate of hippocampal neuron. (a) Representative sEPSC traces. (b) Cumulative probability plots showing change in sEPSC amplitude (left). The histogram shows the difference in amplitude between groups (right). (c). Cumulative probability plots showing change in sEPSC frequency (left). The histogram shows the difference in frequency between groups. (d) Decay time of sEPSC. (e) Rise time of sEPSC. ^∗^*P* < 0.05 and^∗∗^*P* < 0.01, Sham vs. 2VO; ^#^*P* < 0.05, 2VO vs. EA; ^^^*P* < 0.05, EA vs. Non-acu.

The presynaptic membrane releases excitatory neurotransmitters that bind to receptors on the postsynaptic membrane, causing inward currents in it. sEPSCs in resting hippocampal CA1 pyramidal neurons were recorded in whole-cell voltage clamp mode. The brain cell slice clamped the membrane potential to –70 mV, recorded in whole cell mode for 3 min. It responded to changes in excitatory synaptic function and electrical activity of pyramidal neurons in the CA1 region of the hippocampus in each group (Figure 3(a)). Compared with the sham operation group, the frequency and amplitude decreased in the 2VO group (*P* < 0.05). Compared to the 2VO and Non-acu groups, the amplitude and frequency increased in the EA group (Figures 3(b) and 3(c)). Further data analysis revealed a significant decrease in the sEPSC decay and rise times in the 2VO group (*P* < 0.05). EA upregulated the decay and rise times of sEPSCs in the 2VO group (*P* < 0.05). The sEPSC decay and rise times were still increased in the EA group compared to the Non-acu group (*P* < 0.05; Figures 3(d) and 3(e)).

### 3.4. EA Increased the Expression and Phosphorylation Levels of Synapse-Related Proteins in the Hippocampus of VCI Rats

**Figure 4:**
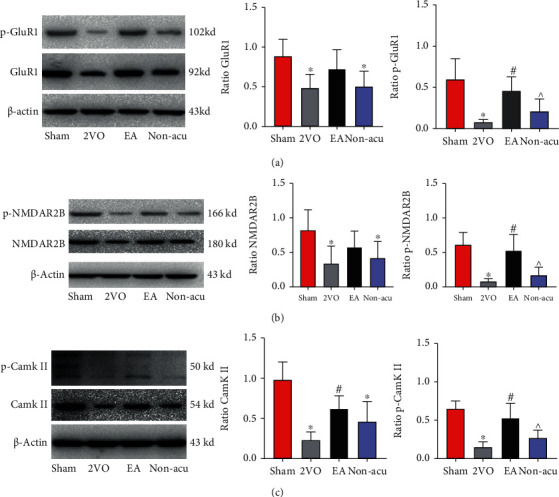
Electroacupuncture improves the expression and phosphorylation level of synapse-related protein in the hippocampus of VCI model rats. (a) Expression levels of GluR1 protein and its phosphorylation levels in the hippocampal region of rats in all groups. (b) Expression levels of NMDAR2B protein and its phosphorylation levels in the hippocampal region of rats in all groups. (c) Expression levels of CaMKII protein and its phosphorylation levels in hippocampal region of rats in all groups. ^∗^*P* < 0.05 and^∗∗^*P* < 0.01, Sham vs. 2VO and Sham vs NA; ^#^*P* < 0.05, 2VO vs EA; ^^^*P* < 0.05, EA vs. Non-acu.

One-way ANOVA revealed a significant main effect of EA. As illustrated in Figure 4. Compared to the sham operation group, GluR1, CaMKII, and NMDAR2B total protein and phosphorylation levels in the 2VO group were significantly reduced (*P* < 0.05). Compared to the 2VO group, GluR1 and NMDAR2B total protein were no statistical difference in EA group, but the phosphorylation levels significantly increased (*P* < 0.05). Compared to the EA group, GluR1, CaMKII, and NMDAR2B phosphorylation levels in the Non-acu group significantly decreased (*P* < 0.05) (Figures 4(a)–4 (c)), suggesting that EA increases the protein phosphorylation levels of NMDAR2B, GluR1, and CaMKII.

## 4. Discussion

The present study revealed that EA at the GV20 and GV24 acupoints for four weeks significantly ameliorated learning and memory deficits and LTP impairments in the hippocampal Schaffer collateral pathway. In addition, it restored the membrane potential of neurons in cerebral hypoperfusion rats. EA is potentially involved in neuroprotective effects, including the regulation of proteins such as NMDAR2B, GluR1, and CaMKII.

Vascular cognitive impairment comprises a spectrum of cerebrovascular diseases with different underlying mechanisms [35]. Clinical syndromes associated with vascular brain diseases are commonly accompanied by cognitive impairment. Neuropsychological examination has shown the hippocampus to be a key region responsible for processing spatial information [36]. The new object experiment before the intervention in this study confirmed that the learning and memory function of rats decreased significantly after modelling. Studies have shown that the cerebral blood flow in the cortex of 2VO model rats reduced to 30–50% of the original level 3 days after the operation, and the cerebral blood flow of the hippocampus reduced by approximately 60% [37]. The study established a transient cerebral ischemia model in rats and performed behavioural tests seven days after surgery. The results showed a gradual decline in the learning and memory function of rats in the 2VO group. Our previous study confirmed that EA at the GV20 and GV24 acupoints effectively alleviates the learning memory ability of rats in a vascular dementia model, but the mechanism through which EA improves learning memory has not been fully elucidated. Here, we investigated the effect of EA on the learning memory function of VCI rats based on our previous study. The results of the Morris water maze test showed that EA shortened the escape latency of VCI rats and increased the number of platform crossings and improve the formation and retrieval of spatial learning and memory. The effect of the on EA group the improvement of learning memory function in VCI rats was significant compared to the Non-acu group, which is consistent with the results of most studies [38]. In the present study, the NA is known as a nonacupoint that is far away from the GV20 and GV24 acupoints to avoid interference with the specificity of the acupuncture point using the side-opening position. Studies on the effects of EA and nonacupoint on cognitive impairment are limited. Clinical and basic studies have mostly set up nonacupoint groups to confirm the specificity of EA therapy [39–41].

LTP provides strong evidence for activity-dependent synaptic plasticity in the higher animal brain and is an ideal model for studying neuronal neural systems. Early studies have shown that the long-term LTP increase is typically 150–200% of the preevoked EPSP amplitude [42]. The results of our study are consistent with these findings. The magnitude of LTP in the CA3–CA1 region increased by approximately 170% in the sham operation group. In another study, LTP in the hippocampal DG region of 2VO rats was recorded using electrophysiological techniques, where selected LTP induction regimen with weak stimulation intensity (200 Hz/time, 10 times in total, 5 s intervals) showed that LTP was 175.5% in the sham group and 135.6% in the 2-VO group [43]. Compared with the LTP induction scheme in this experiment, the LTP induction scheme adopted in this study (100 Hz/times, second times in total, and 30 s intervals) had lower stimulation intensity, which may also be one of the reasons for the relatively weak increase of LTP in the hippocampus of rats in the model group recorded in this experiment. Our LTP results from male rats were similar to LTP results reported in previous studies [44]. Similarly, hippocampal LTP assays have confirmed that rats with chronic cerebral ischemia have significant learning memory dysfunction and impaired synaptic plasticity [45]. In 2VO rats subjected to a two-week EA intervention, in vivo electrophysiological techniques were used to examine the characteristics of LTP in the DG area of the hippocampus after surgery in each group, resulting in LTP damage in the DG area being significantly attenuated in the VCI group and significantly reduced in the EA group [46]. Previous studies have reported that the immediate effect of EA increases LTP in the DG region of the rat hippocampus [47]. Our results showed that the LTP of the EA group was significantly higher than that of the 2VO and NA groups. This observation indicates that acupuncturing GV20 and GV24 could effectively reduce the synaptic plasticity damage of hippocampal neurons in VCI rats, thereby improving their learning and memory functions. I/O curve was used as a measure of synaptic transmission function [48]. This study confirmed that EA can improve the synaptic basic transmission efficiency impairment in the hippocampal CA3-CA1 region caused by VCI model rats. Studies have used six different time intervals of 25, 50, 100, 150, 200, and 250 ms to detect the presynaptic function of rats in each group before detecting hippocampal LTP. PPR is an indicator of synaptic transmission efficiency, changes in which are indicative of presynaptic modulation [49]. The results of this study confirmed no statistically significant differences in the PPR results between the groups, suggesting that the excitatory presynaptic transmission function of neurons in the hippocampus of the rats is not significantly different. Our data indicates a possible regulation of the LTP recovery by EA in the rat models of 2-VO by postsynaptic mechanisms.

Notably, sEPSCs were present in hippocampal neurons. VCI altered several electrophysiological parameters, including sEPSC amplitude, frequency, and rise and decay times. Our study is the first to demonstrate that the frequency and amplitude decreased in the 2VO group. We detected an increased number of excitatory synapses in the EA group, along with an increase in the sEPSC frequency and amplitude [50]. Consistent with the results from previous studies, EA produced an immediate and long-lasting increase in sEPSC frequency and amplitude in an NMDA receptor-dependent manner [51]. Studies detecting the resting state of the hippocampus pyramidal neurons after cerebral ischemia found that the excitatory postsynaptic currents EPSC showed specificity [52]. Another study pointed out that within 24 h after transient complete cerebral ischemia, there was membrane potential of hippocampal neurons, mediated by hyperpolarization, and a reduced synaptic transmission rate, leading to the decrease of the excitatory postsynaptic currents EPSC [53]. It was confirmed that pyramidal neurons in the hippocampal CA1 region are highly sensitive to cerebral ischemia, and sEPSC changes induced by changes in excitatory synaptic transmission may be the internal mechanism leading to neuronal dysfunction after ischemia. The results of this study confirmed that EA upregulated the decay and rise times of sEPSCs in the 2VO group. The faster rise times at mature synapses reflect increased synchrony of multivesicular release, whereas the faster decay appears to reflect changes in the properties of postsynaptic receptors [54].

Studies have shown that activation of GluR1 phosphorylation of AMPA receptor subunit can effectively alleviate LTP damage in the hippocampus of VCI rat brain slices, thus improving VCI learning and memory functioning [55]. Another study showed that phosphorylation of the AMPAR subunit GluR1 effectively attenuated LTP damage in the hippocampal region of brain slices of VCI rats, improving learning and memory functioning in VCI [56]. The results of the present study show that GluR1 and its phosphorylation levels were significantly reduced in the hippocampal region of VCI rats, suggesting that VCI may contribute to synaptic plasticity dysfunction. NMDARs are widely found in the postsynaptic membrane and hippocampal sites of the central nervous system and play a very important role in memory and learning functions [57]. NMDAR2B plays an important role in synaptic plasticity and the formation and storage of spatial working memory [58, 59]. Learning memory is significantly impaired in NMDAR2B knockout rats, while synapses in rats show plasticity changes when NMDAR2B receptors are overexpressed. The mechanism of reduced NMDAR2B/2A expression levels in hippocampal tissue of VCI rats may be related to neuronal death or structural damage induced by hippocampal ischemia and hypoxia [60]. In the present study, western blotting confirmed that hippocampal NMDAR2B and its phosphorylation levels were significantly reduced in VCI rats, whereas EA effectively increased the expression levels of NMDAR2B phosphorylation. CaMKII is highly expressed in neuronal tissues, particularly in the hippocampus of the brain [61], and is a multifunctional protein kinase that regulates neurotransmitter biosynthesis, cytoplasmic division, and synaptic plasticity. Increased CaMKII autophosphorylation is important for long-term sustained enhancement of hippocampal LTP synaptic efficacy [62]. Electron microscopy was used to observe postsynaptic density ultrastructure damage in the hippocampal region of VCI rats. This investigation indicated that the significantly reduced expression and phosphorylation CaMKII in the hippocampal tissue of model rats may possibly lead to changes in synaptic ultrastructure and cognitive function impairment of rats [63]. Phosphorylation plays a fundamental role in most signalling pathways that directly regulate various aspects of protein function. LTP induction results in calcium entry, which activates CaMKII that subsequently translocates to the synapse, where it binds to NMDA receptors and produces potentiation by phosphorylating principal AMPA-type glutamate receptors [64]. In our study, we confirmed that hippocampal CAMKII and its phosphorylation levels were significantly reduced in VCI rats, whereas EA effectively increased CaMKII phosphorylation levels.

In this study, the expression and phosphorylation levels of GluR1, NMDAR, and CaMKII were determined to explore the mechanism through which GV20 and GV24 increased LTP and improved learning and memory function in the hippocampus of VCI rats. However, the formation and maintenance of LTP depend on AMPAR- and NMDAR-mediated neurotransmitter signal transduction, and the specific molecular biological mechanisms of EA that improve the formation and maintenance of LTP remain to be further studied.

## 5. Conclusion

This study is aimed at investigating the effects of acupuncture on behaviours associated with the VCI rat model. The expression of the signalling pathway components of Glu, NMDAR, and CAMKII in the rat hippocampus was also measured. In the VCI rat model, treatment with acupuncture improved behaviours associated with learning and memory, and these effects were associated with changes in the AMPAR, NMDAR, and CaMKII signalling pathways.

EA at the GV20 and GV24 acupoints for four weeks can improve the learning and memory ability of VCI rats by improving synaptic transmission in the hippocampal CA3–CA1 circuit and regulating the expression and phosphorylation levels of postsynaptic membrane receptors NMDAR, AMPAR, and CaMKII. This study provides a better understanding of the molecular mechanisms underlying the traditional use of EA-treated VCI.

## 6. Limitations

Synaptic plasticity is the cellular physiological basis of learning and memory, including the changes in synaptic structure and function. From a synaptic functional plasticity perspective, this study confirmed that electroacupuncture, GV24 and GV24, could enhance LTP intensity in the hippocampal CA3-CA1 region of VCI model rats, improving learning and memory abilities, but lacked synaptic morphological structure and related pathological examination, such as the number, density, and morphology.

## Data Availability

The data used to support the findings of the current study are available from the corresponding author on reasonable request.

## Abbreviations

VCI: Vascular cognitive impairment
WB: Western blotting
I/O: Input-output
2VO: Two-vessel occlusion
HFS: High-frequency stimulation
LTP: Long-term potentiation
PPR: Paired-pulse facilitation ratios
NMDAR: N-Methyl-D-aspartate receptor
AMPAR: *α*-Amino-3hydroxy-5methyl-4isoxazole receptor
fEPSP: Field excitatory postsynaptic plasticity
CaMKII: Ca^2+^-calmodulin-dependent protein kinase II
HIP: Hippocampus
EA: Electroacupuncture
sEPSC: Spontaneous excitatory postsynaptic current.
